# Do Children Who Move Home and School Frequently Have Poorer Educational Outcomes in Their Early Years at School? An Anonymised Cohort Study

**DOI:** 10.1371/journal.pone.0070601

**Published:** 2013-08-05

**Authors:** Hayley A. Hutchings, Annette Evans, Peter Barnes, Joanne Demmler, Martin Heaven, Melanie A. Hyatt, Michelle James-Ellison, Ronan A. Lyons, Alison Maddocks, Shantini Paranjothy, Sarah E. Rodgers, Frank Dunstan

**Affiliations:** 1 Centre for Health Information Research and Evaluation, College of Medicine, Swansea University, Singleton Park, Swansea, United Kingdom; 2 Cochrane Institute of Primary Care and Public Health, Cardiff University, Heath Park, Cardiff, United Kingdom; 3 Department of Public Health, Public Health Wales, St David’s Park, Carmarthen, United Kingdom; 4 Department of Child Health, Abertawe Bromorgannwg University Health Board, Singleton Hospital, Swansea, United Kingdom; University of Sydney, Australia

## Abstract

Frequent mobility has been linked to poorer educational attainment. We investigated the association between moving home and moving school frequently and the early childhood formal educational achievement. We carried out a cohort analysis of 121,422 children with anonymised linked records. Our exposure measures were: 1) the number of residential moves registered with a health care provider, and 2) number of school moves. Our outcome was the formal educational assessment at age 6–7. Binary regression modeling was used to examine residential moves within the three time periods: 0 – <1 year; 1 – <4 years and 4 – <6 years. School moves were examined from age 4 to age 6. We adjusted for demographics, residential moves at different times, school moves and birth related variables. Children who moved home frequently were more likely not to achieve in formal assessments compared with children not moving. Adjusted odds ratios were significant for 3 or more moves within the time period 1 –<4 years and for any number of residential moves within the time period 4–<6 years. There was a dose response relationship, with increased odds ratios with increased frequency of residential moves (2 or more moves at 4–<6 years, adjusted odds ratio 1.16 (1.03, 1.29). The most marked effect was seen with frequent school moves where 2 or more moves resulted in an adjusted odds ratio of 2.33 (1.82, 2.98). This is the first study to examine the relationship between residential and school moves in early childhood and the effect on educational attainment. Children experiencing frequent mobility may be disadvantaged and should be closely monitored. Additional educational support services should be afforded to children, particularly those who frequently change school, in order to help them achieve the expected educational standards.

## Introduction

Moving home is one of the most stressful life events in adults and is often ranked with illness, loss of employment and divorce. In modern society around 50% of children have moved home at least once before their 10th birthday [1]. The removal of a familiar environment and the breakdown of social networks associated with a residential move can result in stress and adjustment problems in children [1], [2], [3].

The emotional and behavioral effects of moves (both school and residential) seem clear. However, the impact of residential moves on educational attainment in children is less apparent. Previous studies have examined the relationship between moving schools and educational outcomes but few have considered the relationship between residential mobility and educational outcomes. Studies that evaluated the effect of school moves largely report a negative impact on educational outcomes, particularly at secondary school level [4], [5], [6]. However parent questionnaire data from the Child Health Supplement to the 1988 National Health Interview Survey [3] indicated that those children between the ages of 7 and 12 who moved home an average or above-average number of times did not have poorer educational attainment if they resided in families in which both biological parents were present. However for children who were in other family structures, any home move was associated with an adverse school life [3]. In contrast to the findings of some United Kingdom (UK) studies [4], [7], [8], an analysis of educational achievement in relation to residential and school mobility illustrated that residential mobility in urban South African children had no detrimental effect on educational outcomes [9]. In some instances, there was even found to be a positive association between relocation and educational outcomes. Most researchers agree that moving (residential and school) affects social relationships that are important to academic achievement [10]. This relationship is complex however, and it has been suggested that most of the negative effect is due to pre-existing differences between movers and non-movers [10].

We analysed data from a total population cohort of children born in Wales, UK, in order to explore the associations between residential and school moves during early childhood and their effects on the educational attainment of children specifically in relation to formal UK National Curriculum Key Stage 1 (ks1) teacher assessments at age 6–7. We examined three time periods to explore the relationship between early residential mobility and educational attainment. We also looked at residential and school mobility during the period of ks1 teaching and the possible influence on educational outcome. We found that there was a significant association between frequent residential and school mobility and early formal educational attainment, particularly where moves occurred in the period immediately prior to educational assessment.

## Materials and Methods

### Ethics Statement

The data held by Health Information Research Unit (HIRU) in the Secure Anonymised Information Linkage (SAIL) System are anonymised and have been obtained with the permission of the relevant Caldicott Guardian/Data Protection Officer; therefore the National Research Ethics Service (NRES) has stated that no ethical review is required. Approval was obtained and granted from the HIRU Information Governance Review Panel (IGRP) to use the SAIL System to answer the specific residential and school moves research questions [11], [12].

### Cohort Development and Composition

The *W*elsh Electronic Cohort for Children (WECC) consists of over 800,000 children born or living within Wales between 1990 and 2008. Individual-level anonymised data on these children were obtained from numerous sources : General Practice data, the Welsh Demographic Service (WDS) which is a continually updated record of children living within Wales, community child health records from the National Community and Child Health Database (NCCHD), births and deaths from the Office for National Statistics (ONS), inpatient and outpatient data from the Patient Episode Database for Wales (PEDW), congenital anomalies from the Congenital Anomaly Register and Information Services (CARIS), free school meal entitlement and environmental data from the National Pupil Database (NPD) and formal educational data from the Pupil Level Annual School Census (PLASC).

WECC was set up within the HIRU at Swansea University, United Kingdom (UK) using the SAIL databank [11]. SAIL is a relational database capable of linking anonymised data at individual and household level across many health and health-related data sets [12]. HIRU uses a robust array of privacy-protecting techniques to overcome the confidentiality and disclosure issues in health related warehousing. As part of the disclosure protection, HIRU does not hold identifiable demographic data (such as names and addresses) but uses anonymised linking fields instead. This means that information can be linked together from different datasets using an Anonymised Linking Field (ALF) and information at the household level can be linked together using Residential Anonymised Linking Fields (RALF) [13], [14]. This unique set-up enables the grouping of individuals living together in the same household and the ability to follow their movement from one residence to the next over time. The smallest geographical units which are held in SAIL are Lower Super Output Areas (LSOAs), which contain approximately 1500 residents. RALFs [13], [14] are assigned for each child which relate to where the child currently lives. A new RALF is assigned when updates are received from health service providers where the patient has indicated a change of address.

### Study Population

Formal educational assessment data from the NPD and PLASC were only available between the years 2003–2008 within WECC. Therefore the study population for this analysis of the cohort was children who were born within Wales between 1 September 1995 and 31 August 2001 (6 academic school years) for whom educational data were available at age 6–7. We excluded stillbirths, deaths before the ks1 assessment date and children with special educational needs (SEN) school action plus or statemented status [15].

### Measure of Exposure

We defined a residential move as “a change of residence that was registered with a health service provider”. The number of residential moves was calculated using data from the Welsh Demographic Survey (WDS) linked to the RALFs as a change in RALF indicated a move. The number of school moves was calculated using the educational NPD and PLASC data.

Key stages in the UK are the four phases into which compulsory education is divided. These are: Key Stage (ks) 1 at age 4/5 to 7, ks2 at age 7–11, ks3 at age 11–14, and ks4 at age 14–16 [16]. Pupils are classified not by individual scores, but by categories. The two main categories are: level achieved or not achieved. Additional categories exist for children who do not fit into these two main categories: absent, disapplied (i.e. not taking the subject), not awarded the level, unable to provide an assessment, and working towards the level assessed by the test. The ks1 statutory assessment for children in language, mathematics and science involves only teacher assessment against attainment targets. An overall assessment (based on achieving the appropriate level within each of the specific subjects) score is provided in addition to the individual subject specific assessments. Our primary outcome measure was *not* achieving in this overall assessment at ks1. This included all the additional categories other than absent, which was coded as a missing value.

### Statistical Analysis

Residential moves were ordered into four categories: 0, 1, 2 and 3 or more [17]. The number of residential moves was examined in each of three time periods (relating to the age of the child): 0 to <1 year; 1 year to <4 years; and 4 years to 6 years. The first period (0 to <1 year) relates to infancy and is perhaps unlikely to impact on school performance and the last period (4 to 6 years) is when the ks1 syllabus is taught and so potentially might have the greatest effect; the middle category consists of the years in between. School moves were ordered into three categories: 0, 1 and 2 or more between Reception year (start age 4 years) and Year 2 (start age 6 years).

Variables other than breastfeeding status had few missing values and only subjects with complete records were included; for breastfeeding about 56% of the records were not available. This was almost certainly for administrative rather than clinical reasons and so we imputed the missing breast feeding data using the methods for multiple imputation described by Little and Rubin [18]. We used binary logistic regression to obtain odds ratios (and 95% confidence intervals) for not achieving in ks1 comparing numbers of residential and school moves and adjusted for pre-defined confounding variables: academic season of birth, eligibility for free school meals, gender, parity, gestational age, maternal age, breast feeding status at birth or 6–8 weeks, and material deprivation measured by the Townsend score [19] of first registered lower super output area (LSOA). To avoid making assumptions of linearity those variables which could take many values were divided into categories and treated as categorical variables. Maternal age was divided into five year bands, with the exception of teenage mothers and those aged at least 40. Townsend deprivation scores were divided into 10 groups, with the first group denoting the least deprived group through to the tenth group denoting the most deprived. Robust standard errors were obtained to account for the clustering of children within schools. We included two-way interaction terms between residential and school moves and tested these based on the change in deviance.

As the frequency of residential moves was likely to be higher for those children in care, modeling of the data was carried out both with and without children in care in the model. As the inclusion of children in care did not affect the outcome of the model, children in care were retained in the analysis. The analysis was carried out using Stata version 11.

### Results

There were 143,869 children in WECC who were born in Wales between 1 September 1995 and 31 August 2001 (6 school years), who met the study criteria and for whom ks1 data on attainment were available. We carried out a complete case analysis on 121,422 children after excluding missing values.

Table 1 shows the joint distribution of the numbers of residential and school moves. Many children moved home, even at a young age. By the age of one, 18,188 (15.0%) of children had moved home at least once; by age four this increased to 54,304 (44.7%) and by age six, 65,892 (54.3%) of children had moved home at least once. By contrast, of those on whom data were available, only 7% of children moved school at least once between reception year and the end of year 2. Perhaps surprisingly many of those moving school did not move home; indeed of those who changed school at least twice, 57% did not move home.

**Table 1:** Number (%) of residential moves according to school moves.

		Frequency of school moves from Reception to end of Year 2						Total	
		0		1		2+			
		(n = 112967)	%	(n = 8067)	%	(n = 408)	%	(n = 121442)	%
Frequency of residentialmoves 0 – <1 year	0	96529	85.4	6430	79.7	295	72.3	103254	85.0
	1	14477	12.8	1397	17.3	87	21.3	15961	13.1
	2	1662	1.5	200	2.5	24	5.9	1886	1.6
	3+	299	0.3	40	0.5	2	0.5	341	0.3
Frequency of residentialmoves 1 – <4 years	0	72658	64.3	4353	54.0	169	41.4	77180	63.6
	1	29869	26.4	2379	29.5	136	33.3	32384	26.7
	2	7659	6.8	874	10.8	55	13.5	8588	7.1
	3+	2781	2.5	461	5.7	48	11.8	3290	2.7
Frequency of residentialmoves 4 – <6 years	0	101489	89.8	6187	76.7	234	57.4	107910	88.9
	1	9250	8.2	1304	16.2	102	25.0	10656	8.8
	2+	2228	2.0	576	7.1	72	17.6	2876	2.4

Table 2 shows the percentages of those achieving ks1 by numbers of residential and school moves and also levels of confounders. The percentage of those achieving ks1 was lower for children who had moved home several times, especially if the moves were when they were of school age. Only 78% of children with at least two residential moves between the ages of 4 and 6 achieved ks1 compared to 87% in those who did not move at that age. The effect of school moves was more marked, with only 64% of those who changed school at least twice achieving ks1. The percentage of not achieving ks1 was higher in boys, in those with free school meals, in children of young mothers, in children not breastfed, in children born prematurely, in children born in the summer and in those living in deprived areas.

**Table 2:** Association between residential moves and educational outcomes adjusted for all variables shown in the table (Odds ratios and 95% CI).

Characteristic	Category	Key Stage 1 exam			Odd Ratio◊ for not achieving KS1 95% CI		
		Total	Not achieved				
		(n = 121422)	(n = 16938)	%			
Frequency of residential moves0 – <1 year	0	103254	13883	13.4	1.00		
	1	15961	2625	16.4	0.99	0.94	1.05
	2	1886	368	19.5	1.07	0.94	1.23
	3+	341	62	18.2	0.87	0.63	1.22
Frequency of residential moves1 – <4 years	0	77180	9834	12.7	1.00		
	1	32384	4818	14.9	1.01	0.96	1.06
	2	8588	1564	18.2	1.07	0.99	1.15
	3+	3290	722	21.9	1.16	1.04	1.29
Frequency of residential moves4 – <6 years	0	107910	14328	13.3	1.00		
	1	10656	1979	18.6	1.08	1.01	1.16
	2+	2876	631	21.9	1.16	1.03	1.29
Frequency of school moves fromReception to end of Year 2	0	112967	14999	13.3	1.00		
	1	8067	1793	22.2	1.54	1.40	1.69
	2+	408	146	35.8	2.33	1.82	2.98
Free School Meal in KS1 year	No	99769	10908	10.9	1.00		
	Yes	21673	6030	27.8	1.81	1.71	1.91
Gender	Male	59937	10037	16.7	1.00		
	Female	61505	6901	11.2	0.74	0.71	0.77
Parity	Nulliparous	51147	5836	11.4	1.00		
	Multiparous	70295	11102	15.8	1.43	1.36	1.50
Maternal age at childbirth	<20 years old	11754	2528	21.5	1.38	1.29	1.48
	20–24	25074	4541	18.1	1.23	1.17	1.30
	25–29	38299	4837	12.6	1.00		
	30–34	31911	3361	10.5	0.90	0.85	0.94
	35–39	12394	1390	11.2	0.94	0.87	1.01
	40+	2010	281	14.0	1.10	0.95	1.28
NCCHD Breast feeding at birthOR at 6–8 weeks∼	No	25115	4487	17.9	1.00		
	Yes	28868	3417	11.8	0.83	0.78	0.88
Townsend deprivation decile of LSOAat birth/within 4 months of birth	1 (least deprived)	10737	692	6.4	1.00		
	2	10194	878	8.6	1.23	1.08	1.40
	3	10517	1072	10.2	1.45	1.27	1.64
	4	10551	1117	10.6	1.43	1.24	1.65
	5	11973	1482	12.4	1.53	1.34	1.75
	6	11613	1583	13.6	1.58	1.38	1.80
	7	13272	1898	14.3	1.60	1.40	1.83
	8	12173	1924	15.8	1.66	1.44	1.91
	9	13803	2553	18.5	1.87	1.64	2.15
	10 (most deprived)	16609	3739	22.5	2.06	1.79	2.38
Gestational age at birth	<28	181	38	21.0	1.30	0.86	1.96
	28–32	1342	285	21.2	1.33	1.14	1.55
	33–36	6839	1221	17.9	1.21	1.12	1.31
	37–40+ weeks	113080	15394	13.6	1.00		
Academic season of Birth	Early (Sept to Dec)	41249	4105	10.0	1.00		
	Middle (Jan to April)	39915	5518	13.8	1.41	1.34	1.48
	Late (May to Aug)	40278	7315	18.2	1.85	1.75	1.94
Special Educational Needs Status*	No special provision	100943	7269	7.2	1.00		
	School Action	20499	9669	47.2	8.95	8.34	9.60

* Statutory Assessment children advised for Special Educational Needs status at the time of KS1 but not yet classified have been classed as no answer (n = 245, 58 achieved, 187 not achieved at KS1).

∼Breastfeeding has been multiply imputed in the model for missing data.

◊adjusted for all variables shown in the table with robust standard errors to account for clustering of children within schools.

The adjusted odds ratios for residential moves before the age of one were not significantly different from 1. There was an increased risk of not achieving ks1 with 3 or more moves between ages 1 and 4, with an OR of 1.16 (95% CI 1.04–1.29). Any residential move after the age of 4 was associated with an increased risk of not achieving ks1; the OR for at least 2 moves was 1.16 (95% CI 1.03–1.29). School moves had a much larger effect, with an OR associated with at least two moves of 2.33 (95%CI 1.82–2.98). Interactions between school and residential moves were explored but did not lead to a significantly improved model fit.

Among the confounders, apart from the special educational needs status, the largest effects were associated with individual and area deprivation (assessed by entitlement to free school moves and Townsend deciles respectively); summer births were also associated with a considerably increased risk of not achieving ks1. It is interesting that an effect of prematurity persists until age 6–7 (see Table S1).

We fitted a further model including interactions between the confounders and between the confounders and the residential and school moves (see Table S2). There were some very small changes in some of the odds ratios concerning residential and school moves. The only interaction to affect interpretation was between one school moves and having an SEN action plan; for children with one school moves the OR with a school action plan was 1.24 (95% CI 1.09–1.41) and for those without was 1.72 (95% CI 1.54–1.91). This model was tested for goodness of fit using the Hosmer-Lemeshow test; the p value was 0.33.

## Discussion

Our study, which examined the effect of frequent residential and school moves on formal educational attainment within early childhood, demonstrated that even a small number of residential moves may have a detrimental effect on educational attainment. Our study also showed that large numbers of children move home frequently, even at a young age. The most marked effect was however seen with frequent school moves. Many of the children in our cohort who experienced a residential move were not subjected to a school move. From the findings of our study, it appears that children who move home frequently will not be as detrimentally affected, in terms of their early educational attainment compared to those children who move school.

Moving home or school may be associated with short term social and health effects in children. In young children symptoms such as refusing to eat, being clingy and becoming aggressive or shy are common. In older school-aged children, moves are more likely to result in changes in sleep patterns, having trouble concentrating and suffering stomach or headaches [20].

The effects of pupils’ moving school on academic success have been researched since World War II [5], [21] and it has been claimed that only a single school move, especially in primary grades, can be traumatic for a child [22]. The authors claim that signs of unresolved psychological pain, such as hitting, bullying, bragging, lying and sometimes withdrawal were demonstrated. This accords with findings from other work, where children’s reactions to moving included fear of parental abandonment, depression and anger [20]. The relationship between moving school and achievement, however is complex, with numerous factors having an influence. For example, children experiencing frequent school moves have been documented as being more often from poorer families with lower levels of educational attainment and from minority ethnic groups [5].

Using data from criterion-referenced test scores in mathematics and language arts, findings from a recent study revealed that students who moved school more frequently performed less well than non-mobile students, with low-income and the level of student mobility within the school also having a negative effect on the academic achievement of the students. A wide range of negative effects from moving school were demonstrated in addition to the lower scores on criterion and norm-referenced tests. These included an increased likelihood of students dropping out from high school, an increase in absenteeism, an increased chance of grade retention and lower citizenship evaluations [5].

Strand et al. have undertaken extensive research to examine the effect of moving school on educational attainment. In a study of primary school children, attainment at the end of key stage 2 (ks2; age 7–11) was examined. It was found that pupils who moved school frequently had lower attainment at ks2. However, the negative association was reduced when confounding factors such as special educational needs and socioeconomic status were accounted for and was eliminated entirely when prior pupil attainment at key stage 1 (ks1) was taken into account [7], [8]. These findings conflict with those of a US study that evaluated the effect of moving school in the elementary grades using meta-analysis. The authors showed that moving school was equivalent to a 3–4 month performance disadvantage in achievement [6]. It is argued that understanding the reason for moving school is key, with the effect of low attainment often being attributable to substantial cultural, educational and social adjustments [8].

Although the effects of frequent residential mobility have been examined in relation to health outcomes [17], there has been limited research that has examined the effect of residential mobility on educational outcomes. Findings from a previous US study highlighted that residential moves were only detrimental in children who did not reside with both biological parents [3]. In a South African study, residential moves were not associated with any detrimental effect on educational attainment [9].

After controlling for confounding factors, our study highlighted a significant effect of frequent residential and school moves on formal educational attainment at ks1, with school moves having the strongest effect.

Our study was conducted using a large retrospective anonymised electronic data cohort. There are strengths and limitations with this methodology. A strength of our study was the large sample size from a whole-population cohort of children with detailed data on the outcome of interest. In addition, the outcomes were collected in a standardised way, and as such this allowed a more rigorous comparison of the data. This design was similar to another recent rigorous study which examined the effect of pre-term birth on ks1 outcomes [23].

One limitation of our study was that there was no information regarding the reasons for moving. Brown et al, have commented that not all children may be detrimentally affected by moving home and for some it could represent a move into a less deprived area or to better housing and therefore have a beneficial effect on their future educational attainment [24]. In addition, in our study, a residential move was defined as the change of address registered with a health care provider. It was recognized that this could be a move next door or to any location within Wales. It relied on the families notifying their health care providers when their address changed. It is likely that some short term stays may not be recorded and would therefore be omitted. In addition some residential moves may not be registered with a health care provider in a timely fashion or there may be delays in receiving information, due to time delays associated with data updates. It is likely therefore that the frequency of residential moves in our study is under reported. A change in residential address may not always result in a change in school, particularly if it resulted in a short move within the same area. The findings from our study would support this, with only a relatively small number of residential moves being associated with a school move in our cohort. Some moves, particularly when they involve a school move too, may be perceived as being more disruptive to the child [10]. Again, our findings would suggest that this was true, as following adjustment for previous residential moves, the children who had the highest odds of not achieving were those who had experienced frequent school moves. Our study did not examine details regarding the distance of the move and for the purposes of our analysis, only the frequency of moves in our pre-specified time periods was examined. The timing of the moves was examined in our models by looking at the age at each residential move.

This study examined the impact of residential and school moves on educational attainment within early childhood. Our findings highlight that both frequent residential and school mobility have a negative effect on early educational attainment, with school moves having the greatest effect. This suggests that there may be inequalities between those children who move home or school more frequently and those who are less mobile. Children experiencing frequent mobility are disadvantaged and at risk of educational non-attainment and should be closely monitored. Additional educational support services should be afforded to children, particularly those who move school more frequently, to address the educational inequalities and to help them achieve the expected educational standards.

Further research is needed to determine whether the effects of frequent residential and school mobility on educational attainment persist in later childhood, adolescence and adulthood. Employing complex dynamic statistical analysis to examine the exact timing and proximity of the residential and school moves in relation to the outcomes of interest would be useful to explore the short and long term effects of moving and how long these effects persist.

## Supporting Information

Table S1 **Confounder variable tables for residential and school moves (complete case analysis; n = 121,442).**
(DOCX)

Table S2 **Association between residential moves and educational outcomes with interactions adjusted for all variables shown in the table (Odds ratios and 95% CI, n = 121442).**
(DOCX)
