# Supplementary material for: Do Children Who Move Home and School Frequently Have Poorer Educational Outcomes in Their Early Years at School? An Anonymised Cohort Study
Source: PLoS One. 2013 Aug 5;8(8):e70601. doi: 10.1371/journal.pone.0070601 (PMC3734306; doi:10.1371/journal.pone.0070601)
Supplement: Table S1 — Confounder variable tables for residential and school moves (complete case analysis; n = 121,442). (DOCX) [file pone.0070601.s001.docx]

Table S1: Confounder variable tables for residential and school moves (complete case analysis; n=121,442): Maternal age at childbirth.

|  | | Maternal Age at Childbirth | | | | | | | | | | | | | |
| --- | --- | --- | --- | --- | --- | --- | --- | --- | --- | --- | --- | --- | --- | --- | --- |
|  |  | <20 years old | | 20-24 | | 25-29 | | 30-34 | | 35-39 | | 40+ | | Total | |
| Characteristic | Category | n | % | n | % | n | % | n | % | n | % | n | % | n | % |
| Frequency of residential moves 0 - < 1 year | 0 | 8134 | 7.9% | 19803 | 19.2% | 33455 | 32.4% | 28715 | 27.8% | 11339 | 11.0% | 1808 | 1.8% | 103254 | 100.0% |
|  | 1 | 3015 | 18.9% | 4578 | 28.7% | 4328 | 27.1% | 2899 | 18.2% | 955 | 6.0% | 186 | 1.2% | 15961 | 100.0% |
|  | 2 | 504 | 26.7% | 576 | 30.5% | 453 | 24.0% | 258 | 13.7% | 81 | 4.3% | 14 | .7% | 1886 | 100.0% |
|  | 3+ | 101 | 29.6% | 117 | 34.3% | 63 | 18.5% | 39 | 11.4% | 19 | 5.6% | 2 | .6% | 341 | 100.0% |
| Frequency of residential moves 1 - < 4 years | 0 | 4573 | 5.9% | 13349 | 17.3% | 25026 | 32.4% | 23116 | 30.0% | 9551 | 12.4% | 1565 | 2.0% | 77180 | 100.0% |
|  | 1 | 4425 | 13.7% | 8095 | 25.0% | 10177 | 31.4% | 7029 | 21.7% | 2296 | 7.1% | 362 | 1.1% | 32384 | 100.0% |
|  | 2 | 1830 | 21.3% | 2557 | 29.8% | 2344 | 27.3% | 1357 | 15.8% | 434 | 5.1% | 66 | .8% | 8588 | 100.0% |
|  | 3+ | 926 | 28.1% | 1073 | 32.6% | 752 | 22.9% | 409 | 12.4% | 113 | 3.4% | 17 | .5% | 3290 | 100.0% |
| Frequency of residential moves 4 - < 6 years | 0 | 8630 | 8.0% | 20844 | 19.3% | 34735 | 32.2% | 29997 | 27.8% | 11797 | 10.9% | 1907 | 1.8% | 107910 | 100.0% |
|  | 1 | 2344 | 22.0% | 3298 | 30.9% | 2851 | 26.8% | 1567 | 14.7% | 513 | 4.8% | 83 | .8% | 10656 | 100.0% |
|  | 2+ | 780 | 27.1% | 932 | 32.4% | 713 | 24.8% | 347 | 12.1% | 84 | 2.9% | 20 | .7% | 2876 | 100.0% |
| Frequency of school moves from Reception to end of Year 2 | 0 | 10235 | 9.1% | 22771 | 20.2% | 35892 | 31.8% | 30359 | 26.9% | 11812 | 10.5% | 1898 | 1.7% | 112967 | 100.0% |
|  | 1 | 1395 | 17.3% | 2178 | 27.0% | 2316 | 28.7% | 1503 | 18.6% | 564 | 7.0% | 111 | 1.4% | 8067 | 100.0% |
|  | 2+ | 124 | 30.4% | 125 | 30.6% | 91 | 22.3% | 49 | 12.0% | 18 | 4.4% | 1 | .2% | 408 | 100.0% |
| Total |  | 11754 | 9.7% | 25074 | 20.6% | 38299 | 31.5% | 31911 | 26.3% | 12394 | 10.2% | 2010 | 1.7% | 121442 | 100.0% |

Table S1 continued: Confounder variable tables for residential and school moves (complete case analysis; n=121,442): Townsend Deprivation deciles of LSOA at birth / within first 4 months of birth.

|  | | Townsend Deprivation deciles of LSOA at birth / within first 4 months of birth | | | | | | | | | | | | | | | | | | | | | |
| --- | --- | --- | --- | --- | --- | --- | --- | --- | --- | --- | --- | --- | --- | --- | --- | --- | --- | --- | --- | --- | --- | --- | --- |
|  |  | 1 - Most wealthy | | 2 | | 3 | | 4 | | 5 | | 6 | | 7 | | 8 | | 9 | | 10 - Most deprived | | Total | |
| Characteristic | Category | n | % | n | % | n | % | n | % | n | % | n | % | n | % | n | % | n | % | n | % | n | % |
| Frequency of residential moves  0 - < 1 year | 0 | 9629 | 9.3% | 9046 | 8.8% | 9235 | 8.9% | 9223 | 8.9% | 10336 | 10.0% | 10004 | 9.7% | 11209 | 10.9% | 10161 | 9.8% | 11206 | 10.9% | 13205 | 12.8% | 103254 | 100.0% |
|  | 1 | 991 | 6.2% | 1006 | 6.3% | 1140 | 7.1% | 1169 | 7.3% | 1453 | 9.1% | 1403 | 8.8% | 1792 | 11.2% | 1777 | 11.1% | 2263 | 14.2% | 2967 | 18.6% | 15961 | 100.0% |
|  | 2 | 105 | 5.6% | 120 | 6.4% | 110 | 5.8% | 136 | 7.2% | 153 | 8.1% | 182 | 9.7% | 227 | 12.0% | 202 | 10.7% | 283 | 15.0% | 368 | 19.5% | 1886 | 100.0% |
|  | 3+ | 12 | 3.5% | 22 | 6.5% | 32 | 9.4% | 23 | 6.7% | 31 | 9.1% | 24 | 7.0% | 44 | 12.9% | 33 | 9.7% | 51 | 15.0% | 69 | 20.2% | 341 | 100.0% |
| Frequency of residential moves  1 - < 4 years | 0 | 7457 | 9.7% | 6931 | 9.0% | 7147 | 9.3% | 7096 | 9.2% | 7851 | 10.2% | 7389 | 9.6% | 8396 | 10.9% | 7486 | 9.7% | 8160 | 10.6% | 9267 | 12.0% | 77180 | 100.0% |
|  | 1 | 2614 | 8.1% | 2475 | 7.6% | 2486 | 7.7% | 2606 | 8.0% | 3087 | 9.5% | 3122 | 9.6% | 3564 | 11.0% | 3358 | 10.4% | 3963 | 12.2% | 5109 | 15.8% | 32384 | 100.0% |
|  | 2 | 507 | 5.9% | 601 | 7.0% | 646 | 7.5% | 597 | 7.0% | 755 | 8.8% | 809 | 9.4% | 940 | 10.9% | 969 | 11.3% | 1188 | 13.8% | 1576 | 18.4% | 8588 | 100.0% |
|  | 3+ | 159 | 4.8% | 187 | 5.7% | 238 | 7.2% | 252 | 7.7% | 280 | 8.5% | 293 | 8.9% | 372 | 11.3% | 360 | 10.9% | 492 | 15.0% | 657 | 20.0% | 3290 | 100.0% |
| Frequency of residential moves 4 - < 6 years | 0 | 9943 | 9.2% | 9344 | 8.7% | 9568 | 8.9% | 9559 | 8.9% | 10758 | 10.0% | 10306 | 9.6% | 11745 | 10.9% | 10674 | 9.9% | 11956 | 11.1% | 14057 | 13.0% | 107910 | 100.0% |
|  | 1 | 653 | 6.1% | 692 | 6.5% | 735 | 6.9% | 796 | 7.5% | 975 | 9.1% | 1023 | 9.6% | 1185 | 11.1% | 1210 | 11.4% | 1412 | 13.3% | 1975 | 18.5% | 10656 | 100.0% |
|  | 2+ | 141 | 4.9% | 158 | 5.5% | 214 | 7.4% | 196 | 6.8% | 240 | 8.3% | 284 | 9.9% | 342 | 11.9% | 289 | 10.0% | 435 | 15.1% | 577 | 20.1% | 2876 | 100.0% |
| Frequency of school moves from Reception to end of Year 2 | 0 | 10266 | 9.1% | 9726 | 8.6% | 9907 | 8.8% | 9926 | 8.8% | 11163 | 9.9% | 10800 | 9.6% | 12323 | 10.9% | 11293 | 10.0% | 12666 | 11.2% | 14897 | 13.2% | 112967 | 100.0% |
|  | 1 | 462 | 5.7% | 449 | 5.6% | 583 | 7.2% | 602 | 7.5% | 780 | 9.7% | 773 | 9.6% | 906 | 11.2% | 839 | 10.4% | 1071 | 13.3% | 1602 | 19.9% | 8067 | 100.0% |
|  | 2+ | 9 | 2.2% | 19 | 4.7% | 27 | 6.6% | 23 | 5.6% | 30 | 7.4% | 40 | 9.8% | 43 | 10.5% | 41 | 10.0% | 66 | 16.2% | 110 | 27.0% | 408 | 100.0% |
| Total | | 10737 | 8.8% | 10194 | 8.4% | 10517 | 8.7% | 10551 | 8.7% | 11973 | 9.9% | 11613 | 9.6% | 13272 | 10.9% | 12173 | 10.0% | 13803 | 11.4% | 16609 | 13.7% | 121442 | 100.0% |

Table S1 continued: Confounder variable tables for residential and school moves (complete case analysis; n=121,442): Special Educational Needs Status.

|  | | Special Educational Needs Status | | | | | |
| --- | --- | --- | --- | --- | --- | --- | --- |
|  |  | No special provision | | School Action | | Total | |
| Characteristic | Category | n | % | n | % | n | % |
| Frequency of residential moves 0 - < 1 year | 0 | 86411 | 83.7% | 16843 | 16.3% | 103254 | 100.0% |
|  | 1 | 12823 | 80.3% | 3138 | 19.7% | 15961 | 100.0% |
|  | 2 | 1447 | 76.7% | 439 | 23.3% | 1886 | 100.0% |
|  | 3+ | 262 | 76.8% | 79 | 23.2% | 341 | 100.0% |
| Frequency of residential moves 1 - < 4 years | 0 | 65083 | 84.3% | 12097 | 15.7% | 77180 | 100.0% |
|  | 1 | 26607 | 82.2% | 5777 | 17.8% | 32384 | 100.0% |
|  | 2 | 6762 | 78.7% | 1826 | 21.3% | 8588 | 100.0% |
|  | 3+ | 2491 | 75.7% | 799 | 24.3% | 3290 | 100.0% |
| Frequency of residential moves 4 - < 6 years | 0 | 90364 | 83.7% | 17546 | 16.3% | 107910 | 100.0% |
|  | 1 | 8368 | 78.5% | 2288 | 21.5% | 10656 | 100.0% |
|  | 2+ | 2211 | 76.9% | 665 | 23.1% | 2876 | 100.0% |
| Frequency of school moves from Reception to end of Year 2 | 0 | 94337 | 83.5% | 18630 | 16.5% | 112967 | 100.0% |
|  | 1 | 6321 | 78.4% | 1746 | 21.6% | 8067 | 100.0% |
|  | 2+ | 285 | 69.9% | 123 | 30.1% | 408 | 100.0% |
| Total | | 100943 | 83.1% | 20499 | 16.9% | 121442 | 100.0% |

Table S1 continued: Confounder variable tables for residential and school moves (complete case analysis; n=121,442): NCCHD Breastfeeding at birth / 6-8 weeks.

|  | | NCCHD Breastfeeding at birth / 6-8 weeks | | | | | | | |
| --- | --- | --- | --- | --- | --- | --- | --- | --- | --- |
|  |  | No | | Yes | | No answer | | Total | |
| Characteristic | Category | n | % | n | % | n | % | n | % |
| Frequency of residential moves 0 - < 1 year | 0 | 21153 | 20.5% | 24887 | 24.1% | 57214 | 55.4% | 103254 | 100.0% |
|  | 1 | 3479 | 21.8% | 3525 | 22.1% | 8957 | 56.1% | 15961 | 100.0% |
|  | 2 | 398 | 21.1% | 383 | 20.3% | 1105 | 58.6% | 1886 | 100.0% |
|  | 3+ | 85 | 24.9% | 73 | 21.4% | 183 | 53.7% | 341 | 100.0% |
| Frequency of residential moves 1 - < 4 years | 0 | 15384 | 19.9% | 18974 | 24.6% | 42822 | 55.5% | 77180 | 100.0% |
|  | 1 | 6955 | 21.5% | 7372 | 22.8% | 18057 | 55.8% | 32384 | 100.0% |
|  | 2 | 1974 | 23.0% | 1843 | 21.5% | 4771 | 55.6% | 8588 | 100.0% |
|  | 3+ | 802 | 24.4% | 679 | 20.6% | 1809 | 55.0% | 3290 | 100.0% |
| Frequency of residential moves 4 - < 6 years | 0 | 21919 | 20.3% | 25966 | 24.1% | 60025 | 55.6% | 107910 | 100.0% |
|  | 1 | 2477 | 23.2% | 2284 | 21.4% | 5895 | 55.3% | 10656 | 100.0% |
|  | 2+ | 719 | 25.0% | 618 | 21.5% | 1539 | 53.5% | 2876 | 100.0% |
| Frequency of school moves from Reception to end of Year 2 | 0 | 23007 | 20.4% | 26732 | 23.7% | 63228 | 56.0% | 112967 | 100.0% |
|  | 1 | 2000 | 24.8% | 2040 | 25.3% | 4027 | 49.9% | 8067 | 100.0% |
|  | 2+ | 108 | 26.5% | 96 | 23.5% | 204 | 50.0% | 408 | 100.0% |
| Total | | 25115 | 20.7% | 28868 | 23.8% | 67459 | 55.5% | 121442 | 100.0% |

Table S1 continued: Confounder variable tables for residential and school moves (complete case analysis; n=121,442): Academic Season of Birth.

|  | | Academic Season of Birth | | | | | | | |
| --- | --- | --- | --- | --- | --- | --- | --- | --- | --- |
|  |  | Early - Sept to Dec | | Middle -  Jan to April | | Late - May to Aug | | Total | |
| Characteristic | Category | n | % | n | % | n | % | n | % |
| Frequency of residential moves 0 - < 1 year | 0 | 34902 | 33.8% | 34005 | 32.9% | 34347 | 33.3% | 103254 | 100.0% |
|  | 1 | 5543 | 34.7% | 5233 | 32.8% | 5185 | 32.5% | 15961 | 100.0% |
|  | 2 | 684 | 36.3% | 583 | 30.9% | 619 | 32.8% | 1886 | 100.0% |
|  | 3+ | 120 | 35.2% | 94 | 27.6% | 127 | 37.2% | 341 | 100.0% |
| Frequency of residential moves 1 - < 4 years | 0 | 26120 | 33.8% | 25260 | 32.7% | 25800 | 33.4% | 77180 | 100.0% |
|  | 1 | 10957 | 33.8% | 10742 | 33.2% | 10685 | 33.0% | 32384 | 100.0% |
|  | 2 | 2991 | 34.8% | 2806 | 32.7% | 2791 | 32.5% | 8588 | 100.0% |
|  | 3+ | 1181 | 35.9% | 1107 | 33.6% | 1002 | 30.5% | 3290 | 100.0% |
| Frequency of residential moves 4 - < 6 years | 0 | 36480 | 33.8% | 35497 | 32.9% | 35933 | 33.3% | 107910 | 100.0% |
|  | 1 | 3789 | 35.6% | 3460 | 32.5% | 3407 | 32.0% | 10656 | 100.0% |
|  | 2+ | 980 | 34.1% | 958 | 33.3% | 938 | 32.6% | 2876 | 100.0% |
| Frequency of school moves from Reception to end of Year 2 | 0 | 38405 | 34.0% | 37181 | 32.9% | 37381 | 33.1% | 112967 | 100.0% |
|  | 1 | 2705 | 33.5% | 2611 | 32.4% | 2751 | 34.1% | 8067 | 100.0% |
|  | 2+ | 139 | 34.1% | 123 | 30.1% | 146 | 35.8% | 408 | 100.0% |
| Total | | 41249 | 34.0% | 39915 | 32.9% | 40278 | 33.2% | 121442 | 100.0% |

Table S1 continued: Confounder variable tables for residential and school moves (complete case analysis; n=121,442): Gestational Age at Birth.

|  | | Gestational Age at Birth | | | | | | | | | |
| --- | --- | --- | --- | --- | --- | --- | --- | --- | --- | --- | --- |
|  |  | <28 weeks | | 28-32 | | 33-36 | | 37-40+ | | Total | |
| Characteristic | Category | N | % | n | % | n | % | n | % | n | % |
| Frequency of residential moves 0 - < 1 year | 0 | 142 | .1% | 1111 | 1.1% | 5773 | 5.6% | 96228 | 93.2% | 103254 | 100.0% |
|  | 1 | 32 | .2% | 209 | 1.3% | 925 | 5.8% | 14795 | 92.7% | 15961 | 100.0% |
|  | 2 | 7 | .4% | 18 | 1.0% | 116 | 6.2% | 1745 | 92.5% | 1886 | 100.0% |
|  | 3+ | 0 | .0% | 4 | 1.2% | 25 | 7.3% | 312 | 91.5% | 341 | 100.0% |
| Frequency of residential moves 1 - < 4 years | 0 | 103 | .1% | 839 | 1.1% | 4238 | 5.5% | 72000 | 93.3% | 77180 | 100.0% |
|  | 1 | 61 | .2% | 377 | 1.2% | 1859 | 5.7% | 30087 | 92.9% | 32384 | 100.0% |
|  | 2 | 11 | .1% | 90 | 1.0% | 528 | 6.1% | 7959 | 92.7% | 8588 | 100.0% |
|  | 3+ | 6 | .2% | 36 | 1.1% | 214 | 6.5% | 3034 | 92.2% | 3290 | 100.0% |
| Frequency of residential moves 4 - < 6 years | 0 | 155 | .1% | 1170 | 1.1% | 6004 | 5.6% | 100581 | 93.2% | 107910 | 100.0% |
|  | 1 | 14 | .1% | 130 | 1.2% | 660 | 6.2% | 9852 | 92.5% | 10656 | 100.0% |
|  | 2+ | 12 | .4% | 42 | 1.5% | 175 | 6.1% | 2647 | 92.0% | 2876 | 100.0% |
| Frequency of school moves from Reception to end of Year 2 | 0 | 168 | .1% | 1242 | 1.1% | 6341 | 5.6% | 105216 | 93.1% | 112967 | 100.0% |
|  | 1 | 13 | .2% | 90 | 1.1% | 480 | 6.0% | 7484 | 92.8% | 8067 | 100.0% |
|  | 2+ | 0 | .0% | 10 | 2.5% | 18 | 4.4% | 380 | 93.1% | 408 | 100.0% |
| Total | | 181 | .1% | 1342 | 1.1% | 6839 | 5.6% | 113080 | 93.1% | 121442 | 100.0% |

Table S1 continued: Confounder variable tables for residential and school moves (complete case analysis; n=121,442): Gender.

|  | | Gender | | | | | |
| --- | --- | --- | --- | --- | --- | --- | --- |
|  |  | Male | | Female | | Total | |
| Characteristic | Category | n | % | n | % | n | % |
| Frequency of residential moves 0 - < 1 year | 0 | 50942 | 49.3% | 52312 | 50.7% | 103254 | 100.0% |
|  | 1 | 7905 | 49.5% | 8056 | 50.5% | 15961 | 100.0% |
|  | 2 | 916 | 48.6% | 970 | 51.4% | 1886 | 100.0% |
|  | 3+ | 174 | 51.0% | 167 | 49.0% | 341 | 100.0% |
| Frequency of residential moves 1 - < 4 years | 0 | 38264 | 49.6% | 38916 | 50.4% | 77180 | 100.0% |
|  | 1 | 15844 | 48.9% | 16540 | 51.1% | 32384 | 100.0% |
|  | 2 | 4198 | 48.9% | 4390 | 51.1% | 8588 | 100.0% |
|  | 3+ | 1631 | 49.6% | 1659 | 50.4% | 3290 | 100.0% |
| Frequency of residential moves 4 - < 6 years | 0 | 53313 | 49.4% | 54597 | 50.6% | 107910 | 100.0% |
|  | 1 | 5201 | 48.8% | 5455 | 51.2% | 10656 | 100.0% |
|  | 2+ | 1423 | 49.5% | 1453 | 50.5% | 2876 | 100.0% |
| Frequency of school moves from Reception to end of Year 2 | 0 | 55718 | 49.3% | 57249 | 50.7% | 112967 | 100.0% |
|  | 1 | 4022 | 49.9% | 4045 | 50.1% | 8067 | 100.0% |
|  | 2+ | 197 | 48.3% | 211 | 51.7% | 408 | 100.0% |
| Total | | 59937 | 49.4% | 61505 | 50.6% | 121442 | 100.0% |

Table S1 continued: Confounder variable tables for residential and school moves (complete case analysis; n=121,442): Free School Meal taken in KS1 year.

|  | | Free School Meal taken in KS1 year | | | | | |
| --- | --- | --- | --- | --- | --- | --- | --- |
|  |  | No | | Yes | | Total | |
| Characteristic | Category | n | % | n | n | % | n |
| Frequency of residential moves 0 - < 1 year | 0 | 86600 | 83.9% | 16654 | 16.1% | 103254 | 100.0% |
|  | 1 | 11676 | 73.2% | 4285 | 26.8% | 15961 | 100.0% |
|  | 2 | 1274 | 67.6% | 612 | 32.4% | 1886 | 100.0% |
|  | 3+ | 219 | 64.2% | 122 | 35.8% | 341 | 100.0% |
| Frequency of residential moves 1 - < 4 years | 0 | 66336 | 85.9% | 10844 | 14.1% | 77180 | 100.0% |
|  | 1 | 25435 | 78.5% | 6949 | 21.5% | 32384 | 100.0% |
|  | 2 | 5993 | 69.8% | 2595 | 30.2% | 8588 | 100.0% |
|  | 3+ | 2005 | 60.9% | 1285 | 39.1% | 3290 | 100.0% |
| Frequency of residential moves 4 - < 6 years | 0 | 90609 | 84.0% | 17301 | 16.0% | 107910 | 100.0% |
|  | 1 | 7367 | 69.1% | 3289 | 30.9% | 10656 | 100.0% |
|  | 2+ | 1793 | 62.3% | 1083 | 37.7% | 2876 | 100.0% |
| Frequency of school moves from Reception to end of Year 2 | 0 | 93795 | 83.0% | 19172 | 17.0% | 112967 | 100.0% |
|  | 1 | 5745 | 71.2% | 2322 | 28.8% | 8067 | 100.0% |
|  | 2+ | 229 | 56.1% | 179 | 43.9% | 408 | 100.0% |
| Total | | 99769 | 82.2% | 21673 | 17.8% | 121442 | 100.0% |
