# Supplementary material for: Do Children Who Move Home and School Frequently Have Poorer Educational Outcomes in Their Early Years at School? An Anonymised Cohort Study
Source: PLoS One. 2013 Aug 5;8(8):e70601. doi: 10.1371/journal.pone.0070601 (PMC3734306; doi:10.1371/journal.pone.0070601)
Supplement: Table S2 — Association between residential moves and educational outcomes with interactions adjusted for all variables shown in the table (Odds ratios and 95% CI, n = 121442). (DOCX) [file pone.0070601.s002.docx]

Table S2: Association between residential moves and educational outcomes with interactions adjusted for all variables shown in the table (Odds ratios and 95% CI, n=121442)

| Characteristic | Category | Odd Ratio◊  for not achieving KS1 | 95% CI | |
| --- | --- | --- | --- | --- |
| Frequency of residential moves  0 - < 1 year | 0 | 1.00 |  |  |
|  | 1 | 0.99 | 0.93 | 1.04 |
|  | 2 | 1.06 | 0.93 | 1.20 |
|  | 3+ | 0.89 | 0.65 | 1.21 |
| Frequency of residential moves  1 - < 4 year | 0 | 1.00 |  |  |
|  | 1 | 1.01 | 0.96 | 1.05 |
|  | 2 | 1.06 | 0.99 | 1.14 |
|  | 3+ | 1.17 | 1.05 | 1.29 |
| Frequency of residential moves  4 - < 6 years | 0 | 1.00 |  |  |
|  | 1 | 1.08 | 1.01 | 1.15 |
|  | 2+ | 1.15 | 1.03 | 1.28 |
| Frequency of school moves from Reception to end of Year 2 | 0 | 1.00 |  |  |
|  | 1 | 1.72 | 1.54 | 1.91 |
|  | 2+ | 2.33 | 1.65 | 3.30 |
| Special Educational Needs (SEN) Status* | No special provision | 1.00 |  |  |
|  | School Action | 18.94 | 15.19 | 23.61 |
| Interaction: Frequency of school moves from Reception to end of Year 2 and SEN Status | 1 school move X School Action | 0.72 | 0.62 | 0.84 |
|  | 2+ school moves X School Action | 0.95 | 0.48 | 1.88 |
| Gender | Male | 1.00 |  |  |
|  | Female | 0.65 | 0.62 | 0.69 |
| Interaction: Gender and SEN status | Female X School Action | 1.36 | 1.26 | 1.48 |
| Parity | Nulliparous |  |  |  |
|  | Multiparous | 1.58 | 1.48 | 1.68 |
| Interaction: Parity and SEN status | Multiparous X School Action | 0.77 | 0.70 | 0.84 |
| Free School Meal (FSM) in KS1 year | No | 1.00 |  |  |
|  | Yes | 3.25 | 2.41 | 4.39 |
| Interaction: FSM in KS1 year and SEN status | FSM in KS1 year X School Action | 0.70 | 0.64 | 0.77 |
| Maternal age at childbirth | <20 years old | 1.83 | 1.65 | 2.03 |
|  | 20-24 | 1.39 | 1.29 | 1.51 |
|  | 25-29 | 1.00 |  |  |
|  | 30-34 | 0.84 | 0.78 | 0.91 |
|  | 35-39 | 0.88 | 0.79 | 0.98 |
|  | 40+ | 0.99 | 0.79 | 1.23 |
| Interaction: Maternal age and SEN status | <20 years old X School Action | 0.70 | 0.61 | 0.80 |
|  | 20-24 X School Action | 0.81 | 0.73 | 0.90 |
|  | 30-34 X School Action | 1.10 | 0.99 | 1.22 |
|  | 35-39 X School Action | 1.07 | 0.92 | 1.23 |
|  | 40+ X School Action | 1.23 | 0.90 | 1.68 |
| Interaction: Maternal age and FSM in KS1 year | <20 years old X Yes FSM | 0.74 | 0.65 | 0.85 |
|  | 20-24 X Yes FSM | 0.89 | 0.79 | 1.00 |
|  | 30-34 X Yes FSM | 1.18 | 1.03 | 1.35 |
|  | 35-39 X Yes FSM | 1.22 | 1.02 | 1.46 |
|  | 40+ X Yes FSM | 1.09 | 0.74 | 1.61 |
| NCCHD Breast feeding at birth OR at 6-8 weeks~ | No | 1.00 |  |  |
|  | Yes | 0.83 | 0.78 | 0.88 |
| Gestational age at birth | <28 | 1.24 | 0.82 | 1.89 |
|  | 28-32 | 1.33 | 1.14 | 1.55 |
|  | 33-36 | 1.20 | 1.12 | 1.30 |
|  | 37-40+ weeks | 1.00 |  |  |

Table S2 continued: Association between residential moves and educational outcomes with interactions adjusted for all variables shown in the table (Odds ratios and 95% CI, n=121442)

| Characteristic | Category | Odd Ratio◊  for not achieving KS1 | 95% CI | |
| --- | --- | --- | --- | --- |
| Academic season of Birth | Early (Sept to Dec) | 1.00 |  |  |
|  | Middle (Jan to April) | 1.48 | 1.40 | 1.58 |
|  | Late (May to Aug) | 2.14 | 2.01 | 2.28 |
| Interaction: Academic season of Birth and SEN status | Middle (Jan to April) X School Action | 0.88 | 0.79 | 0.97 |
|  | Late (May to Aug) X School Action | 0.70 | 0.64 | 0.77 |
| Townsend deprivation decile of LSOA at birth /within 4 months of birth 4 - < 6 years | 1 (least deprived) | 1.00 |  |  |
|  | 2 | 1.33 | 1.10 | 1.60 |
|  | 3 | 1.60 | 1.33 | 1.93 |
|  | 4 | 1.56 | 1.28 | 1.90 |
|  | 5 | 1.80 | 1.50 | 2.18 |
|  | 6 | 1.72 | 1.42 | 2.07 |
|  | 7 | 1.87 | 1.56 | 2.25 |
|  | 8 | 1.95 | 1.60 | 2.37 |
|  | 9 | 2.23 | 1.86 | 2.68 |
|  | 10 (most deprived) | 2.53 | 2.09 | 3.06 |
| Interaction: Townsend deprivation deciles and SEN status | 2 X School Action | 0.82 | 0.64 | 1.06 |
|  | 3 X School Action | 0.80 | 0.62 | 1.02 |
|  | 4 X School Action | 0.80 | 0.62 | 1.02 |
|  | 5 X School Action | 0.71 | 0.56 | 0.89 |
|  | 6 X School Action | 0.75 | 0.59 | 0.94 |
|  | 7 X School Action | 0.70 | 0.56 | 0.88 |
|  | 8 X School Action | 0.71 | 0.57 | 0.90 |
|  | 9 X School Action | 0.67 | 0.53 | 0.84 |
|  | 10 (most deprived) X School Action | 0.69 | 0.56 | 0.87 |
| Interaction: Townsend deprivation deciles FSM in KS1 year | 2 X Yes FSM | 0.86 | 0.59 | 1.25 |
|  | 3 X Yes FSM | 0.77 | 0.55 | 1.08 |
|  | 4 X Yes FSM | 0.80 | 0.57 | 1.13 |
|  | 5 X Yes FSM | 0.66 | 0.48 | 0.90 |
|  | 6 X Yes FSM | 0.80 | 0.58 | 1.10 |
|  | 7 X Yes FSM | 0.66 | 0.49 | 0.89 |
|  | 8 X Yes FSM | 0.64 | 0.47 | 0.88 |
|  | 9 X Yes FSM | 0.67 | 0.50 | 0.91 |
|  | 10 (most deprived) X Yes FSM | 0.61 | 0.45 | 0.82 |

| * Statutory Assessment children advised for Special Educational Needs status at the time of KS1 but not yet classified have been classed as no answer (n=245, 58 achieved, 187 not achieved at KS1).  ~ Breastfeeding has been multiply imputed in the model for missing data  ◊ adjusted for all variables shown in the table with robust standard errors to account for clustering of children within schools | | |  |  |
| --- | --- | --- | --- | --- |
|  |  |  |  |  |
|  |  |  |  |  |
|  | | |  |  |
